# Draft Genome Sequence of *Saccharomonospora* sp. Strain LRS4.154, a Moderately Halophilic Actinobacterium with the Biotechnologically Relevant Polyketide Synthase and Nonribosomal Peptide Synthetase Systems

**DOI:** 10.1128/genomeA.00392-17

**Published:** 2017-05-25

**Authors:** Scarlett Alonso-Carmona, Blanca Vera-Gargallo, Rafael R. de la Haba, Antonio Ventosa, Horacio Sandoval-Trujillo, Ninfa Ramírez-Durán

**Affiliations:** a Faculty of Medicine, Autonomous University of the State of Mexico, Toluca, Mexico; b Departament of Microbiology and Parasitology, Faculty of Pharmacy, University of Sevilla, Seville, Spain; c Departament of Biological Systems, Metropolitan Autonomous University–Xochimilco, Mexico City, Distrito Federal, Mexico

## Abstract

The draft genome sequence of *Saccharomonospora* sp. strain LRS4.154, a moderately halophilic actinobacterium, has been determined. The genome has 4,860,108 bp, a G+C content of 71.0%, and 4,525 open reading frames (ORFs). The clusters of *PKS* and *NRPS* genes, responsible for the biosynthesis of a large number of biomolecules, were identified in the genome.

## GENOME ANNOUNCEMENT

Actinobacteria represent a group of microorganisms that are prolific in producing secondary metabolites (1). However, the biotechnological potential of its halophilic representatives remains unexplored (2). The ability to synthesize secondary metabolites is closely associated with the identification of the polyketide synthase (PKS) and nonribosomal peptide synthetase (NRPS) gene clusters in an actinobacterial genome sequence. These genes are responsible for the synthesis of antibiotics, biosurfactants, siderophores, immunosuppressants, antitumoral and antiviral agents, and biomolecules with important medical and biotechnological applications (3, 4).

The strain LRS4.154 is a moderately halophilic actinobacterium, isolated from a soil sample from Laguna del Rosario in Oaxaca, Mexico. Based on 16S rRNA gene sequence comparisons, the strain LRS4.154 could be affiliated with the genus *Saccharomonospora*, with *Saccharomonospora azurea* and *Saccharomonospora xinjiangensis* being the closest relatives, both sharing 98.4% 16S rRNA gene sequence similarity. The aim of this work was to sequence the draft genome of the strain LRS4.154 in order to unravel its potential for future applications in biomedical areas.

The draft genome sequence of *Saccharomonospora* sp. LRS4.154 was accomplished using a whole-genome shotgun strategy (5) on an Illumina HiSeq 2500 platform (2 × 100-bp paired-end) (STAB VIDA, Portugal), yielding 12,366,802 reads and 846.63 Mbp. The genome assembly was performed using the software Velvet version 1.2.10 (6) and resulted in 13 contigs (≥1,000 bp) with an *N*_50_ value of 731,563. The NCBI Prokaryotic Genome Annotation Pipeline (7) was used to identify open reading frames (ORFs) and provide a functional annotation of protein-coding genes, as well as other functional genome units.

The draft genome is estimated to contain 4,860,108 bp, with a G+C content of 71.0%. The reported coding density is 90.3%, with 0.916 genes per kbp. A total of 4,525 putative ORFs were predicted, with an average size of 985 bp, as well as two noncoding RNA (ncRNA), four rRNA (one 16S rRNA, one 23S, and two 5S rRNA), and 47 tRNA genes.

Analysis of the genome confirms the presence of six *PKS* and two *NRPS* gene clusters. These bioinformatic data suggest the potential of the halophilic actinobacterial strain LSR4.154 to produce secondary metabolites or unknown metabolites of PKS and NRPS origin. This is the first halophilic *Saccharomonospora* strain reported until now having those capabilities.

### Accession number(s).

This whole-genome shotgun project has been deposited in DDBJ/ENA/GenBank under the accession no. MWIH00000000. The version described in this paper is the first version, MWIH01000000.

## References

[1] Raja A, Prabakaran P 2011 Actinomycetes and drug-an overview. Am J Drug Discov Dev 1:75–84. doi:10.3923/ajdd.2011.75.84.

[2] Hamedi J, Mohammadipanah F, Ventosa A 2013 Systematic and biotechnological aspects of halophilic and halotolerant actinomycetes. Extremophiles 17:1–13. doi:10.1007/s00792-012-0493-5. 23129307

[3] Roongsawang N, Lim SP, Washio K, Takano K, Kanaya S, Morikawa M 2005 Phylogenetic analysis of condensation domains in the nonribosomal peptide synthetases. FEMS Microbiol Lett 252:143–151. doi:10.1016/j.femsle.2005.08.041. 16182472

[4] Jenifer JS, Donio MB, Michaelbabu M, Vincent SG, Citarasu T 2015 Haloalkaliphilic *Streptomyces* spp. AJ8 isolated from solar salt works and its’ pharmacological potential. AMB Express 5:143. doi:10.1186/s13568-015-0143-2. 26307214 PMC4549370

[5] Fleischmann RD, Adams MD, White O, Clayton RA, Kirkness EF, Kerlavage AR, Bult CJ, Tomb JF, Dougherty BA, Merrick JM, McKenney K, Sutton GG, FitzHugh W, Fields CA, Gocayne JD, Scott JD, Shirley R, Liu LI, Glodek A, Kelley JM, Weidman JF, Phillips CA, Spriggs T, Hedblom E, Cotton MD, Utterback T, Hanna MC, Nguyen DT, Saudek DM, Brandon RC, Fine LD, Fritchman JL, Fuhrmann JL, Geoghagen NS, Gnehm CL, McDonald LA, Small KV, Fraser CM, Smith HO, Venter JC 1995 Whole-genome random sequencing and assembly of *Haemophylus influenzae* Rd. Science 269:496–512. doi:10.1126/science.7542800. 7542800

[6] Zerbino DR, Birney E 2008 Velvet: algorithms for de novo short read assembly using de Brijin graphs. Genome Res 18:821–829. doi:10.1101/gr.074492.107. 18349386 PMC2336801

[7] Tatusova T, DiCuccio M, Badretdin A, Chetvernin V, Ciufo S, Li W 2013 Prokaryotic genome annotation pipeline. *In* Beck J, Benson D, Coleman J, Hoeppner M, Johnson M, Maglott D, Mizrachi I, Morris R, Ostell J, Pruitt K, Rubinstein W, Sayers E, Sirotkin K, Tatusova T (ed), The NCBI handbook, 2nd ed National Center for Biotechnology Information, Bethesda, MD.

